# Statistical Inferences Applying Non-Parametric Data on Cyanobacterial Investigations: Contributions to Water Quality and New Trends under Global Changes on Portuguese Freshwater Ecosystems

**DOI:** 10.3390/toxins14090638

**Published:** 2022-09-15

**Authors:** Cristiana Moreira, Ana Matos, Aldo Barreiro, Cidália Gomes, Vitor Vasconcelos, Agostinho Antunes

**Affiliations:** 1 CIIMAR/CIMAR—Interdisciplinary Centre of Marine and Environmental Research, University of Porto, Terminal de Cruzeiros do Porto de Leixões, Av. General Norton de Matos, s/n, 4450–208 Porto, Portugal; 2 Department of Biology, Faculty of Sciences, University of Porto, Rua do Campo Alegre, 4169-007 Porto, Portugal

**Keywords:** *Microcystis aeruginosa*, *Raphidiopsis raciborskii*, *Planktothrix agardhii*, logistic regression, air temperature

## Abstract

Cyanobacteria are a bloom-forming ancient group of photosynthetic prokaryotes. A rise in temperature is a major contributor to its massive proliferation, namely on freshwater ecosystems, with social and economic impacts. Thus, reliable and cost-effective tools can permit the fast surveillance and assessment of temperature effects on potentially toxic cyanobacteria distribution and impacts. The occurrence of three potentially toxic cyanobacteria species was assessed on seven sampling points across three sampling years. Moreover, the association between the occurrence of those cyanobacteria species with climate change events was addressed. Here, we combined molecular and statistical methods to study the impacts of temperature on the occurrence of three globally occurring cyanotoxin-producing cyanobacteria species—*Microcystis aeruginosa* (microcystins), *Raphidiopsis raciborskii* (cylindrospermopsins and saxitoxins) and *Planktothrix agardhii* (microcystins and saxitoxins). Samples were collected on seven European temperate freshwater systems located on the North and Centre regions of Portugal, across three distinct sampling years with distinct ranges of air temperature. Data support that *M. aeruginosa* is still a common inhabitant of Portuguese freshwater ecosystems and a new trend was found on *R. raciborskii* recent invasion and establishment on the colder north ecosystems of Portugal. Additionally, the highest frequency of detection of both cyanobacteria was associated with warmer years. *P. agardhii* also revealed a new trend, being reported for the first time on North and Centre Regions of Portugal, however with no statistical relation with air temperature, demonstrating a higher ecological fitness. Distinct profiles of the statistical analysis on the three tested cyanobacteria species contribute to deepen the studies on other species as well as of our analyzed species on a global level. This assessment may help to anticipate possible repercussions on water quality and public health due to most probable alterations on cyanotoxins profile given the ecological fitness established among air temperature and PCR detection of potentially toxic cyanobacteria.

## 1. Introduction

Cyanobacteria are photosynthetic microorganisms that are among the most ancient living species in Earth [1,2]. They are an important source of oxygen and exist in filamentous and unicellular forms [3]. Their distribution covers aquatic, aerial and terrestrial habitats [3]. These microorganisms interfere in the functioning of ecosystems affecting biodiversity through the production of dense blooms. Eutrophication (excess of nutrients) of water systems, mainly due to anthropogenic activities, is responsible for the increasing incidence of these blooms, along with the rise in water temperature [4]. Production of secondary metabolites is a common phenomenon in cyanobacteria, many with toxic properties such as hepatotoxic, neurotoxic, cytotoxic, dermatoxic and genotoxic or as promoters of cancer [5,6,7]. In general, these compounds have been associated with cases of human and animal poisoning as well as death, due to the ingestion or contact with contaminated waters [8]. In this sense, three routes of exposure have been attributed to cyanobacteria contamination—inhalation, oral and dermal. In ecosystems that are intensively used for irrigation, drinking, washing, water provision and/or recreational purposes, cyanobacteria occurrence as well as of their toxins is mandatory to improve water quality. Globally occurring cyanobacteria such as *Microcystis aeruginosa* (Order Chroococcales), *Raphidiopsis raciborskii* (Order Nostocales) and *Planktothrix agardhii* (Order Oscillatoriales) are common inhabitants of freshwater ecosystems worldwide. These are associated with the production of cyanotoxins such as microcystins (*M. aeruginosa* and *P. agardhii*), cylindrospermopsins (*R. raciborskii*) or saxitoxins (*R. raciborskii* and *P. agardhii*) [9,10,11,12]. In this sense, methods to allow their fast, cost-effective and reliable evaluation are mandatory for an ecological assessment as well as in water quality, evaluation of new trends or to establish the statistical significance of the relationship between detection and environmental parameters such as air temperature. Therefore, it becomes fundamental to apply early warning methods in these microorganisms, as molecular techniques. These have been applied in cyanobacterial research since the early 1990s. They are faster and more accurate, allowing the detection, in diverse types of matrices (soil, pure cultures, water), of genes associated with the cyanobacteria, providing important empirical data of their presence. Polymerase Chain Reaction (PCR) is among the most commonly used techniques consisting of the amplification of a specific DNA sequence. Several genetic markers have been applied in cyanobacterial detection such as DNA gyrase subunit β in *M. aeruginosa* and RNA polymerase *rpoC1* in *R. raciborskii* and *P. agardhii* [13,14,15].

In Portugal, *M. aeruginosa*, *R. raciborskii* and *P. agardhii* have been previously documented. *M. aeruginosa* is a common inhabitant in Portuguese freshwater ecosystems and well associated with microcystin production and bloom formation [15]. The *M. aeruginosa* Portuguese population has been described as possessing a total of 15 genotypes and a southern population structure that is distinct from the rest of the country [16]. *R. raciborskii* is an invasive species found predominantly in the South Region, where it is known to form blooms [17]. Recently, it was found to invade a lagoon in the Centre Region (Vela Lagoon) without, however, forming any blooms. To date, in the North Region of Portugal, this cyanobacterium species is still unreported. Despite, in Portuguese freshwater systems, *R. raciborskii* having no associated toxicity in terms of cylindrospermopsin or saxitoxin production, it has been associated with an unidentified metabolite that possesses unknown toxicity [18]. *P. agardhii* was found to occur only in the South Region of Portugal, forming blooms in coastal waters [15,19] but without any known toxicity.

The main objectives of this study were to assess the presence of these three potentially toxic cyanobacteria species (*M. aeruginosa*, *R. raciborskii* and *P. agardhii*) directly from environmental samples (water) retrieved from seven European temperate freshwater ecosystems located in the North (River Tâmega, Torrão reservoir, Porto City Park Lakes 1, 2 and 3) and Centre Regions (Mira and Vela Lagoons) of Portugal across three funded sampling years (2012, 2013 and 2017), all with distinct ranges of air temperature. The chosen sampling sites carry a great impact since they are commonly used for the practice of recreational activities, agricultural use or as a source of potable water. Furthermore, the impact of environmental variables (air temperature), the implications of climate change events (heat waves) and the new trends of these three cyanobacteria species were also assessed.

## 2. Results

### 2.1. Air Temperature

The maximum values of air temperature varied between 27 and 30 °C in 2012, between 25 and 28 in 2013, and between 28 and 35 °C in 2017 according to IPMA annual reports. These corresponded to an increase in bloom occurrence that was higher in 2012 and in 2017 in contrast to 2013, when no blooms were found in any of the sampling sites and dates. Additionally, comparing both warmer years, 2017 had the highest temperatures registered in Portugal, described as an exceptionally warm year with the occurrence of two heat waves. The analysis of the interannual variation of temperature demonstrates an increase in temperature in all sampling months, among the three sampling years (Supplementary Figure S1).

### 2.2. Bloom Occurrence

Blooms were visually inspected in 2012 at River Tâmega, Torrão Reservoir and Vela Lagoon. However, in 2017, blooms were visually inspected in all sampling locations with the exception of Lake 3 of Porto City Park. In 2013, no blooms were observed in any of the sampling locations.

### 2.3. Isolation

**Figure 1:**
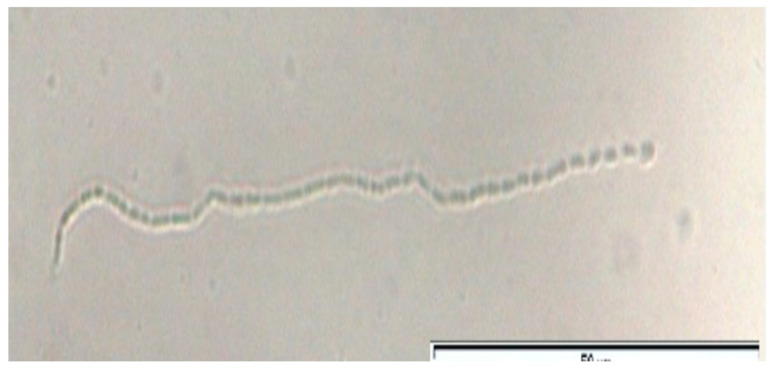
*R. raciborskii* isolate from Lake 2 of Porto City Park.

Strains of *M. aeruginosa* and *R. raciborskii* were isolated. The isolate of *M. aeruginosa* belonged to the sampling site Tâmega River of the month of August of 2013. The isolate of *R. raciborskii* belonged to the sampling site of Porto City Park Lake 2 of the month of June of 2013 (Figure 1). It was not possible to obtain any isolate of *P. agardhii* in any of the sampled years; however, it was observed under the microscope.

### 2.4. M. aeruginosa

**Figure 2:**
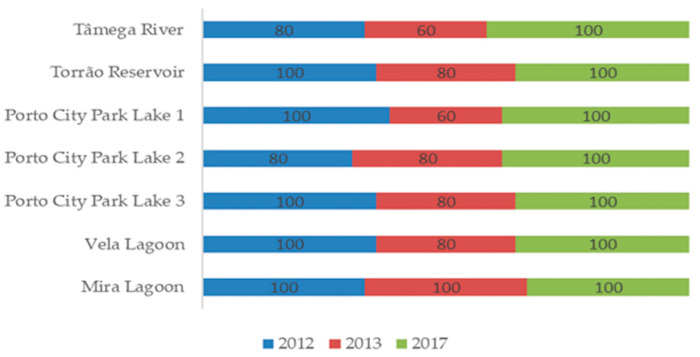
Graphical representation of the percentage of the frequency of detection of *M. aeruginosa* in each sampling site per sampling year.

In Figure 2 is the graphical representation of the percentage of the average detection frequency of *M. aeruginosa* in all sampling sites per sampling year. Results show that the highest detection frequency belonged to the years 2012 and 2017—this last in turn showed the highest detection frequency, with all sampling sites having in total 100% of detection, i.e., all samples tested positive for the presence of this cyanobacterium species. In contrast, 2013 had the lowest detection frequency of all the three sampling years. In terms of geographical distribution, the Centre Region showed the highest average detection frequency when compared to the North Region sampling sites in the same sampling period. Overall, a low seasonal variability in the distribution of *M. aeruginosa* was found to occur in North and Centre Portuguese freshwater ecosystems. In 2012, *M. aeruginosa* was not detected in Tâmega River and in Lake 2 of Porto City Park in May and July, respectively. Mira Lake was the only sampling site where the detection frequency of *M. aeruginosa* was 100% in all sampling years. According to the logistic regression, *M. aeruginosa* showed a significant effect of the factor “year” (*p* < 0.01), the probability of detection in the warmer years (2012 and 2017) being higher in comparison to the less warm year (2013) and with no significant differences between regions (*p* = 0.059) (Table 1).

### 2.5. R. raciborskii

**Figure 3:**
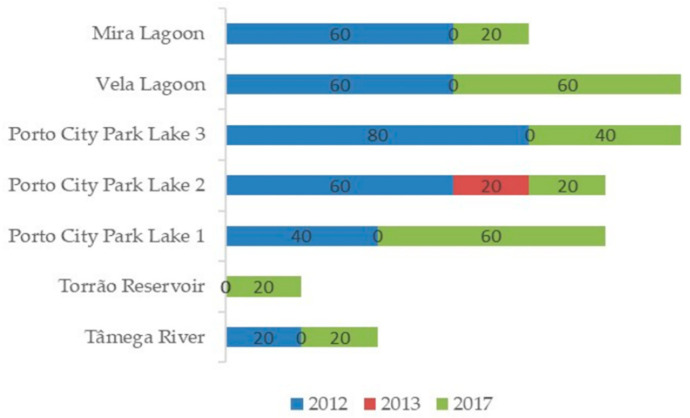
Graphical representation of the percentage of the frequency of detection of *R. raciborskii* in each sampling site per sampling year.

In Figure 3 is the graphical representation of *R. raciborskii* percentage of the average detection frequency of each sampling site per sampling year. Data revealed that the highest detection frequency of this cyanobacterium species, in all three sampling years, was in 2012 and 2017. In 2013, there was only one positive result belonging to the sample Lake 2 of Porto City Park of May. In terms of geographical distribution in all sampling years the highest detection frequency belonged to the samples in the Centre Region. In 2012, *R. raciborskii* was detected in all the sampling sites in at least one of the sampled months with the exception of Torrão Reservoir. In particular, this species had the highest detection frequency in the sample belonging to Lake 3 of Porto City Park. In 2017, Torrão Reservoir had its first detection in all the three sampling years of this cyanobacterium species and this belonged to the sample of September. Overall, Lake 3 of Porto City Park and Vela Lagoon had the highest detection frequency of all the three sampled years. According to the logistic regression, the factor ‘year’ had an influence on the probability of detection for *R. raciborskii* (*p* < 0.001), showing a higher detection frequency in the warmer years (specially 2017) in comparison to the less warm year (2013) with no significant differences between regions (*p* = 0.37) (Table 1).

### 2.6. P. agardhii

**Figure 4:**
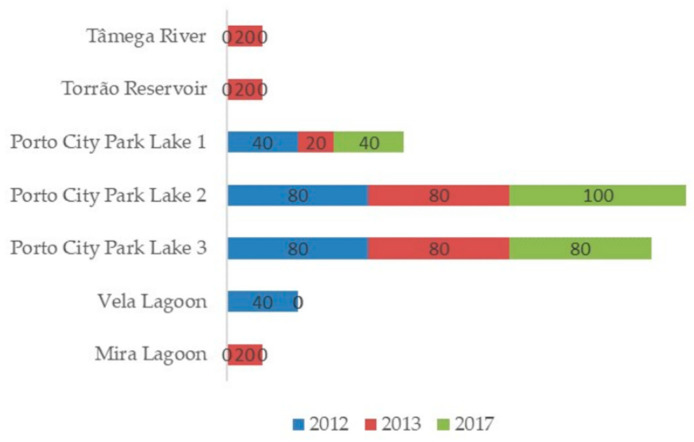
Graphical representation of the percentage of the frequency of detection of *P. agardhii* in each sampling site per sampling year.

In Figure 4 is the graphical representation of *P. agardhii* percentage of the average detection frequency of each sampling site per sampling year. As the logistic regression shows, the detection frequency was not much different among sampling years (*p* = 0.92) and between regions (*p* = 0.18) with the statistical analysis supporting this evidence (Table 1). Overall, the highest detection frequency occurred in the three lakes of Porto City Park. River Tâmega and Torrão Reservoir had only one positive sample of all the three analyzed sampling years, and this belonged to the year 2013. North Region ecosystems had the highest detection frequency of all the three sampling years in comparison to the Centre Region ecosystems with Mira Lagoon having only one positive (June of 2013) and Vela Lagoon with two positives (August and September of 2012). Average detection frequencies show that *P. agardhii* had higher values than *R. raciborskii* and lower than *M. aeruginosa* in both North and Centre Regions. Analyzing all the sampling months in 2012, the presence of this species was mainly recorded in May, August and September. However, in 2013, *P. agardhii* detection occurred mainly in June and it was found everywhere with the exception of Vela Lagoon. In 2017, it was found only in the samples belonging to Lakes 1, 2 and 3 of Porto City Park. In addition, PCR experiments on *mcyA* (microcystins) resulted negative on all isolates.

## 3. Discussion

The main aim of this study was to analyze the dynamics of three globally occurring cyanobacteria species—*M. aeruginosa*, *R. raciborskii* and *P. agardhii*—on seven European temperate freshwater ecosystems. For this, we combined molecular techniques (PCR) with statistical analysis to determine the possible implications of climate change events (heat waves, air temperature), water quality and new trends. Over a three-year sampling period, the sampling years 2012 and 2017 registered higher bloom occurrence compared with the year 2013, these data being in accordance with the registered values of air temperature. In fact, 2017 was an exceptionally warmer year in Portugal, with two heat waves during the sampling period, which may have contributed to the rise in cyanobacterial blooms and the extension of these throughout the sampling period. Therefore, this could have contributed to the rise in cyanobacterial detection frequency in all the three studied cyanobacteria species. Additionally, bloom composition in 2012 was mainly attributed to *Microcystis* sp. while, in 2017, its composition shifted being attributed to either *Microcystis* alone or a mixture of *Microcystis* with other filamentous strains belonging either to the genus *Chrysosphorum* or *Dolichospermum*. These direct observations suggest that the rise in air temperature, in Portugal in the recent years, has favored the increase in other cyanobacteria species, as early records revealed *Microcystis* dominating the bloom composition in Portugal [16]. In contrast, *R. raciborskii* and *P. agardhii* did not form any blooms in both sampled regions during the three-year sampling period.

Of all the analyzed cyanobacteria species in this study, *M. aeruginosa* showed higher detection frequencies in both North and Centre Regions when compared to the other two filamentous cyanobacteria species studied. *M. aeruginosa* was detected in all the sampling sites and across all months of the three-year sampling period. In Portugal, it has been previously demonstrated that *M. aeruginosa* is widely distributed in Portuguese freshwater ecosystems being well described as a bloom-forming cyanobacterium species [16,20]. These data are in agreement with that of van Gremberghe and others (2011) [21] that demonstrated in a worldwide study, applying also molecular tools (Denaturing Gradient Gel Electrophoresis), that *M. aeruginosa* has a cosmopolitan distribution [21]. The logistic regression performed in our study showed that *M. aeruginosa* was detected in a higher amount in the warmer years. In this sense, this study demonstrated that the highest detection frequencies of *M. aeruginosa* are statistically related to the higher ranges of air temperature. This phenomenon could be in part also attributed to the occurrence of blooms of *Microcystis* in both warmer years in higher frequency than in the less warm year (2013). In this sense, *M. aeruginosa* is still the most common bloom-forming cyanobacterium species in Portuguese freshwater ecosystems.

Regarding *R. raciborskii*, the data showed that its average detection frequency in all the three sampling years and sampling sites was the lowest. *R. raciborskii* is well associated with blooms in the South Region of Portugal [17]. Recently, it was found in a lagoon in the Centre Region (Vela Lagoon) without, however, forming any blooms. In both regions, cylindrospermopsins values were detected and attributed to *Chrysosporum* sp. instead of *R. raciborskii* [17,22]. In the North Region of Portugal, its occurrence was, until this study, not detected becoming this the first report of *R. raciborskii* presence in the North Region ecosystems of Portugal either through the application of molecular methods or through the culturing of a *R. raciborskii* isolate. Additionally, the expansion of *R. raciborskii* in the Centre Region was observed. Apart from the initially described Vela Lagoon, *R. raciborskii* was detected in Mira Lagoon in two of the three sampling years. This cyanobacterium is considered biogeographically an invasive species and in Portugal its invasion and subsequent establishment was observed in this study. This new trend of *R. raciborskii* can be explained by the presence of akinetes that confers more resistance to harsh conditions such as lower temperatures. The fact that *R. raciborskii* was found in North Portuguese freshwater ecosystems could be explained through dispersion either by migratory birds, humans, fish or river courses [23] or through the possibility they were initially present and with the rise in air temperatures its detection frequency increased, allowing determination of its presence in surface waters [24]. Nonetheless, the frequencies of detection were significantly higher in warmer years. However, and since between these there is a period of five years, it is well understood that *R. raciborskii* is currently a well-established cyanobacterium species in North Portuguese freshwater ecosystems. In this sense, climate change may be playing an important role in the emergence of *R. raciborskii* in northern Portuguese freshwater ecosystems. However, further surveillance will highlight if *R. raciborskii* will be a common cyanobacterium species in the North and Centre Regions of Portugal or if heat waves become frequent there could be the probable appearance of blooms and toxic forms of *R. raciborskii* in these two regions since in the South bloom occurrence is already a common phenomenon. These results also highlight the importance of surveilling *R. raciborskii* in European freshwaters systems since climate change phenomenon through heat waves have affected this continent shedding concern in the toxicity of this bloom forming cyanobacterium species with particular emphasis in the colder regions of Europe.

In accordance with what was determined in *R. raciborskii*, detection of *P. agardhii* was not as frequent as the detection of *M. aeruginosa* in the studied freshwater ecosystems. However, its average detection frequency did not show a remarkable change between the three sampling years in contrary to *R. raciborskii* and *M. aeruginosa*. To date, the occurrence of *P. agardhii* had only been reported in the South Region of Portugal [15]. However, *Planktothrix* sp. has been already detected in the lakes of Porto City Park (North Region) [25]. The detection of *P. agardhii* in this study in both North and Centre Regions of Portugal therefore constitutes a first report. While we could not succeed in obtaining any isolate of *P. agardhii*, DNA sequencing of a positive amplicon and its observation under microscope of phytoplankton samples confirmed its presence (data not shown). Statistical analyses showed that the dispersion of *P. agardhii* to North and Centre Regions carries no association with the factor air temperature with the average detection frequency being similar in all the three sampling years. However, other atmospheric variables as low precipitation amount or dissemination vectors such as migratory birds, winds or humans could have also contributed to the emergence of *P. agardhii* on both North and Centre Regions of Portugal. This filamentous cyanobacterium can grow in a wide range of temperatures from 10 to 31 °C [12], which favors its expansion also being frequently found in turbid waters [26]. There are many factors responsible for the increase in water turbidity such as the input of agriculture and sewage residue in water systems as well as the occurrence of blooms [27], which may explain the higher detection frequency of *P. agardhii* in relation to *R. raciborskii*. The occurrence of blooms dominated by *M. aeruginosa* could have led to an increase in the water turbidity favoring the growth of *P. agardhii* since *R. raciborskii* prefers less turbid waters [26]. Though no bloom occurrence has yet been attributed to *R. raciborskii* and *P. agardhii*, the high detection frequency, namely associated with warmer years in *R. raciborskii* and without no association with the rise in temperature in *P. agardhii* sheds concern that, in the future, blooms of these two cyanobacteria species may occur in the sampled regions. This could be more probable if ecosystems carry even greater anthropogenic pressures (such as tourism activities) and if there is an increase in hot abnormal events in Portugal such as those that occurred in 2017 causing a shift in bloom composition.

In this study, the first detection and dispersion of two relevant filamentous cyanobacterium species (*R. raciborskii* and *P. agardhii*) and the widespread presence of *M. aeruginosa* were confirmed, reinforcing the application of molecular methodologies as an auxiliary tool in water quality (shaping cyanotoxins profile given cyanobacteria dynamics) and in the assessment of new trends (dispersion). PCR analysis proved to be a suitable tool in the monitoring since the phytoplankton evaluation correlated well with the PCR detection meaning that bloom-forming species were detected in each bloom sample in our PCR analysis. The existence of many positives, mainly of *M. aeruginosa* (a bloom-forming species), permits encouraging the application of PCR in the surveillance of cyanobacteria as a reliable tool. In contrast, the application of other molecular techniques such as quantitative PCR could have resulted in the failure in the enumeration of *M. aeruginosa* for instance due to the lack of primers for such. Despite this, microscopic observation and genera identification conducted on our water and bloom samples surpass the regulatory phytoplankton count, therefore lacking statistical correlation on this data on our studied variables.

Simultaneously with the statistical analyses undertaken here as a first study in the application of non-parametric data, we showed that *M. aeruginosa* and *R. raciborskii* occurrence is associated with air temperature in contrast to *P. agardhii* that showed more resistance to these environmental alterations which may reveal a higher ecological fitness regarding the climate parameter analyzed (air temperature).

In future, further studies will highlight how climate change events such as those that occurred recently in Portugal can be responsible for the occurrence of other relevant cyanobacterium species such as those belonging to the genera *Chrysosphorum* sp. or *Dolichospermum* sp. Finally, the application of non-parametric data (PCR) in a statistical inference (logistic regression) contributed to the understanding of the effects of climate change events in potentially toxic cyanobacteria occurrence with relevant implications in water quality since these may shape cyanotoxins profile. Additionally, new trends were observed particularly in a three-year sampling period with a time frame of five years fostering the gathering and analysis of data, particularly during longer periods of time. Additionally relevant is the continuous monitoring of cyanobacteria, particularly for toxigenic strains in order to assess possible alterations on cyanotoxicity production since on our samples show a shift in bloom composition was observed and, without toxicity being evaluated, this highlights concern in terms of public health as well as water quality also interfering with the current monitoring programs, where for instance *R. raciborskii* and *P. agardhii* were to date uncharacterized in the North and Center Regions of Portugal. Finally, the statistical inferences retrieved by our data may reveal new dynamics in the occurrence of *M. aeruginosa*, *R. raciborskii* and *P. agardhii* in Portuguese freshwater ecosystems regarding the meteorological parameter air temperature, which may be relevant in the detection of cyanotoxin-producing cyanobacteria and in the early warning of blooms and cyanotoxins.

## 4. Conclusions

*M. aeruginosa*, *R. raciborskii* and *P. agardhii* are three cyanotoxin-producing globally occurring cyanobacterium species. Application of cost-effective tools (non-parametric data) in statistical inference monitoring programs can evaluate the influence of ecological variables such as air temperature in toxic cyanobacteria occurrence. This study applied molecular tools (PCR) to screen for the presence of the abovementioned cyanobacteria species in Portuguese temperate freshwater ecosystems. Through the application of a statistical method, new correlations were established between the percentage of the detection frequencies of each cyanobacteria species and environmental data (air temperature) that is intrinsically associated with the superficial water temperature and climate change. A combination of molecular and statistical methods highlighted that *M. aeruginosa* and *R. raciborskii* detection seems associated with air temperature, while *P. agardhii* apparently carries no relation with these alterations, revealing a higher ecological fitness that should be continuously investigated. Our data also reveal the continuous dominance of *M. aeruginosa* in Portugal and a new invasive trend of *R. raciborskii* to North Portuguese ecosystems, as well as the first detection of *P. agardhii* in the studied regions. This study contributes to the improvement of reliable methodologies in the environmental surveillance of cyanobacteria in light of the current climate conditions it is facing also fostering the evaluation of newer perspectives when choosing other ecological variables to improve water quality, public health as well as contributing to climate change prediction in cyanobacteria research. Finally, this study reinforces the need to alter monitoring campaigns in Portuguese freshwater systems since new trends were observed with most probable implications on water quality (blooms and cyanotoxins).

## 5. Materials and Methods

### 5.1. Sampling

**Figure 5:**
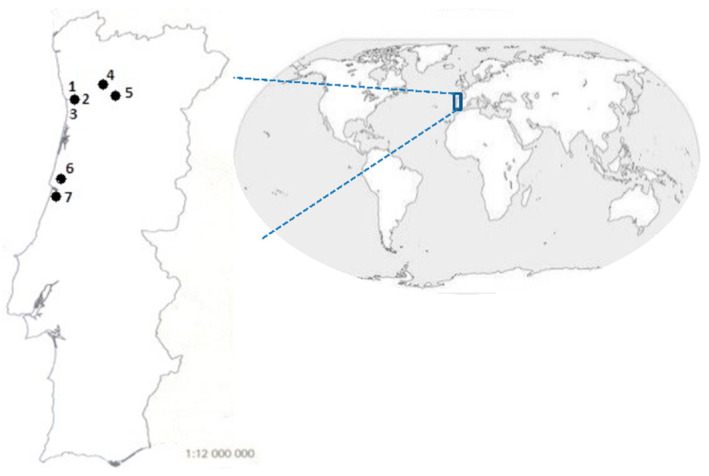
Map of Portugal with the sampling sites marked: (1) City Park of Porto—Lake 1 (41°10′07.1″ N, 8°40′20.5″ W), (2) City Park of Porto—Lake 2 (41°10′04.5″ N, 8°40′25.6″ W), (3) City Park of Porto—Lake 3 (41°10′01.5″ N, 8°40′39.8″ W), (4) Tâmega River (Marco de Canaveses) (41°11′45.9″ N, 8°09′38.2″ W), (5) Torrão Reservoir (41°05′45.7″ N, 8°15′15.4″ W), (6) Mira Lake (40°26′29.8″ N, 8°45′07.5″ W) and (7) Vela Lake (40°16′23.9″ N, 8°47′35.1″ W). Sampling sites 1, 2, 3, 4 and 5 are from the North region while sites 6 and 7 are from the Centre region of Portugal.

A total of seven freshwater systems located in Portugal were monthly surveyed from May to September of 2012, 2013 and 2017 (n = 105). Five systems were located in the North Region (Lakes 1, 2 and 3 of Porto City Park, Tâmega River at Marco de Canaveses and Torrão Reservoir) (Figure 5) all with eutrophic status except Lake 3 on Porto City Park (mesotrophic) [Law Decree 152/97 Diário da República n°139/1997 Série I-A de 1997-06-19 2959–2967; Moreira et al., 2020]. The other two systems were located in the Center Region of Portugal (Mira Lagoon and Vela Lagoon) (Figure 5) both with eutrophic status [Law Decree 152/97 Diário da República n° 139/1997 Série I-A de 1997-06-19 2959–2967] (Figure 1). Water samples were collected from the near shore up to a maximum of 2 L and from the surface. In August and September of 2012, sampling at Torrão Reservoir was not allowed since it became inaccessible due to constructions near this sampling site. Besides water samples, a plankton net with a 55 μm-mesh was used to collect phytoplankton for further isolation of *M. aeruginosa*, *R. raciborskii* and *P. agardhii*. Bloom occurrence was registered at each sampling site and date. Atmospheric temperatures were obtained from the Portuguese Institute of the Sea and Atmosphere (IPMA; Instituto Português do Mar e da Atmosfera) website in each annual report (www.ipma.pt, accessed on 1 November on 2017) (Suplemmentary Figure S1). All samples were maintained in refrigerated conditions until arrival to the laboratory and processed within 24 h after sampling.

### 5.2. Sample Processing

Water samples were immediately filtered upon arrival to the laboratory using a vacuum filtration system and glass-microfiber filters (grade MG C, 47 mm diameter, 1.2 μm porosity) (Munktell®, Sweden). Filtered biomass was maintained at −20 °C until further DNA extraction.

### 5.3. Isolation and Culturing

Phytoplankton samples, collected in each sampling site, were visualized under an optic microscope (Leica DMLB, Germany) and isolated through the micromanipulation technique using a Pasteur pipette. Both colonial and filamentous cyanobacteria strains belonging to *M. aeruginosa*, *R. raciborskii* and *P. agardhii* were isolated and transferred into culture tubes supplemented of 5 mL of Z8 culture medium to promote cyanobacterial growth [28]. Isolated microorganisms were maintained in culture under 25 °C, with a photoperiod of 14 h:10 h light-dark and 25 μEm2s of light intensity without aeration. Cultures were transferred to 50 mL culture flasks with filter caps (Orange Scientific, Braine-l’Alleud, Belgium) containing Z8 culture medium and maintained in the same growth conditions. Cultures were visualized in an Olympus BX41 optic microscope coupled with an Olympus DP72 photograph machine and using Olympus Cell^B software for image acquisition.

### 5.4. DNA Extraction

DNA was extracted from the filtered biomass with the PureLink™ Genomic DNA Kit (Invitrogen, Carlsbad, CA, USA) following the protocol for Gram-negative bacteria. DNA was eluted in 50 μL of elution buffer. Genomic DNA was stored at −20 °C until further molecular analysis.

### 5.5. PCR Amplification

All PCR reactions were performed using the GoTaq® DNA polymerase (Promega, Madison, WI, USA) in a volume of 20 μL containing 1 × PCR buffer, 2.5 mM MgCl_2_, 250 μM of each deoxynucleotide triphosphate, 10 pmol of each primer and 0.5 U of Taq DNA polymerase. To detect the presence of cyanobacteria, the 16S rRNA gene was amplified using the primer pairs 27F/809R and 740F/1494R with an initial denaturation at 95 °C for 2 min and 35 cycles at 92 °C for 20 s, 50 °C for 30 s and 72 °C for 60 s [29,30]. A *Microcystis aeruginosa* strain LEGE 00063 was used as a positive control in the PCR experiments. PCR amplification of a fragment of *M. aeruginosa* DNA gyrase subunit β was achieved using the primer pair gyrF/gyrR. PCR conditions consisted in an initial denaturation at 94 °C for 3 min followed by 35 cycles at 94 °C for 1 min, 60 °C for 1 min and 72 °C for 30 s [14]. A *Microcystis aeruginosa* strain LEGE 00063 was used as a positive control in the PCR experiments. PCR amplification of a fragment of the *R. raciborskii* RNA polymerase rpoC1 gene was achieved using the primer pair cyl2/cyl4. PCR conditions consisted in an initial denaturation at 95 °C for 2 min and 35 cycles at 95 °C for 90 s, 45 °C for 30 s and 72 °C for 50 s [13]. A *R. raciborskii* strain LEGE 95046 was used as a positive control in the PCR experiments. PCR amplification of a fragment of the RNA polymerase rpoC1 *P. agardhii* gene was achieved using the primer pair rpoC1_Plank_F271/rpoC1_Plank_R472. PCR conditions consisted in an initial denaturation at 94 °C for 3 min and 35 cycles at 94 °C for 20 s, 58 °C for 20 s and 72 °C for 20 s [15]. A *P. agardhii* strain LEGE 00280 was used as a positive control in the PCR experiments. Microcystin *mcyA* PCR amplification was performed using the primer pair *mcya*-cd1F/*mcya*-cd1R with the following PCR conditions: an initial denaturation at 95 °C for 2 min and 35 cycles at 95 °C for 90 s, 56 °C for 30 s and 72 °C for 50 s [31]. A *Microcystis aeruginosa* microcystin-producing strain LEGE 00063 was used as a positive control in all the microcystins PCR experiments. All PCR reactions were carried out in a TProfessional Thermocycler (Biometra, Göttingen, Germany) and on a Bio-Rad MyCyclerTM (Bio-Rad, Hercules, CA, USA). PCR products were visualized on a 1% agarose gel electrophoresis.

### 5.6. Statistical Analysis

For the comparison of the frequency of positives between years and regions, a logistic regression was performed with each species, using sample outcome (positive/negative) as dependent variable and year and region as fixed factors. ‘Lake’ was initially included as random factor, but since none of the mixed effects models were better in terms of AIC (Akaike information criterion) than the fixed effects models, the fixed models were selected as the best ones. These analyses were performed with R software, applying the packages stats, lme4 and car (R Core Team 2014). The underlying hypothesis to be tested was to determine if in warmer years (2012 and 2017) the average on the detection frequency was different of the less warm year (2013) in each of the three analyzed cyanobacteria species (*M. aeruginosa*, *R. raciborskii* and *P. agardhii*).

## Figures and Tables

**Table 1:** Results from logistic regression applying R version 4.2.1 software (Vienna, Austria). Chi-square (***χ***^2^), degree of freedom and *p*-values are described.

Analysis of Deviance				
*M. aeruginosa*	**Factor**	***χ^2^***	***df***	***p***
	Year	12.38	2	<0.01
	Region	3.58	1	0.059
	Rescaled model coefficients for Year: Intercept (2012) = 0.97; 2013 = 0.30; 2017 = 1			
*R. raciborskii*	Year	21.55	2	<0.001
	Region	0.81	1	0.37
	Rescaled model coefficients for Year: Intercept (2012) = 0.54; 2013 = 0.033; 2017 = 0.38			
*P. agardhii*	Year	0.16	2	0.92
	Region	1.83	1	0.18

## Data Availability

Not applicable.

## Supplementary Materials

The following supporting information can be downloaded at: https://www.mdpi.com/article/10.3390/toxins14090638/s1, Figure S1: Annual temperature variation. Average of the maximum temperatures observed per each sampling point and year. Porto refers to Lake City Centre 1-3, Figueira refers to Vela Lagoon, Dunas refers to Mira Lagoon and Luzim refers to Torrão and Marco Reservoir.
