# Supplementary material for: Statistical Inferences Applying Non-Parametric Data on Cyanobacterial Investigations: Contributions to Water Quality and New Trends under Global Changes on Portuguese Freshwater Ecosystems
Source: Toxins (Basel). 2022 Sep 15;14(9):638. doi: 10.3390/toxins14090638 (PMC9506200; doi:10.3390/toxins14090638)

Supplementary Figure S1 – Annual temperature variation. Average of the maximum temperatures observed per each sampling point and year. Porto refers to Lake City Centre 1-3, Figueira refers to Vela Lagoon, Dunas refers to Mira Lagoon and Luzim refers to Torrão and Marco Reservoir.

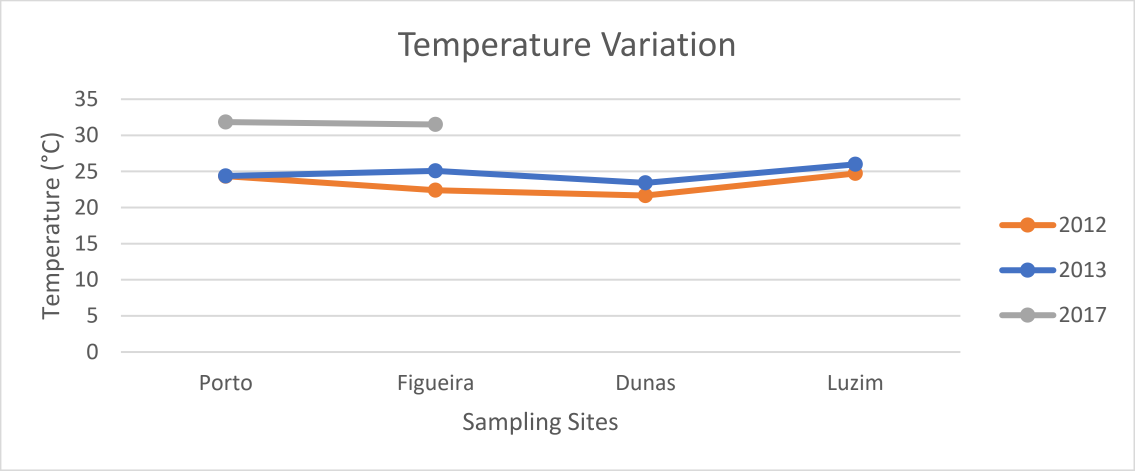

Supplement: Supplementary file 1 [file toxins-14-00638-s001.zip › toxins-1806559-supplementary.pdf]
